# Exploring the potential role of microbiota and metabolites in acute exacerbation of chronic obstructive pulmonary disease

**DOI:** 10.3389/fmicb.2024.1487393

**Published:** 2024-10-16

**Authors:** Yanmin Shi, Jianya Yang, Tao Tian, Suyun Li, Yang Xie

**Affiliations:** ^1^National Regional Traditional Chinese Medicine (Lung Disease) Diagnosis and Treatment Center, The First Affiliated Hospital of Henan University of Chinese Medicine, Zhengzhou, China; ^2^Collaborative Innovation Center for Chinese Medicine and Respiratory Diseases Co-Construction by Henan Province and Education Ministry of P.R. China, Henan University of Chinese Medicine, Zhengzhou, China; ^3^Henan International Joint Laboratory of Evidence-based Evaluation for Respiratory Diseases, Henan Province Clinical Research Center for Respiratory Diseases, The First Affiliated Hospital of Henan University of Chinese Medicine, Zhengzhou, China

**Keywords:** COPD, AECOPD, respiratory microbiota, metabolite, maker

## Abstract

The acute exacerbation of chronic obstructive pulmonary disease seriously affects the respiratory system function and quality of life of patients. This study employed 16S rRNA sequencing and metabolomics techniques to analyze the respiratory microbiota and serum metabolites of COPD and AECOPD patients. The results showed that the microbial diversity in the respiratory tract of AECOPD patients was significantly lower than that of COPD patients, and the relative abundance of Bacteroidetes, *Prevotella* and *Neisseria* in the respiratory tract of AECOPD patients was significantly lower than that of COPD patients. However, the relative abundance of *Haemophilus_D*, *Veillonella_A* and *Pseudomonas_E*, in AECOPD patients was significantly higher than that of COPD patients, and the ability of respiratory microbiota in AECOPD patients to participate in alanine metabolism was significantly lower than that of COPD patients. Metabolome results further revealed that the serum alanine levels in AECOPD patients were significantly lower than those in COPD patients, and these differential metabolites were mainly involved in linoleic acid metabolism, protein digestion and absorption and regulation of lipolysis in adipocytes. In summary, the structural characteristics of respiratory microbiota in COPD and AECOPD patients are different from those in healthy populations, and their microbiota diversity decreases and microbial community structure and function will also undergo changes when acute exacerbations occur. In addition, the predicted microbial community function and metabolomics results indicate that the onset of AECOPD is mainly related to energy and amino acid metabolism disorders, especially alanine metabolism.

## 1 Introduction

Chronic obstructive pulmonary disease (COPD) is a heterogeneous lung disease characterized by chronic respiratory symptoms such as dyspnea, cough, and sputum production, which are related to abnormalities in the airway and alveoli and present as persistent and progressively worsening airflow obstruction (Devadoss et al., 2019). When its symptoms worsen rapidly, it is called acute exacerbation of chronic obstructive pulmonary disease (AECOPD), which is a critical event in COPD management. It has serious adverse effects on individual health status and disease progression, often requiring additional treatment, and is an independent risk factor for death in the vast majority of COPD patients (Ritchie and Wedzicha, 2020; Christenson et al., 2022). In clinical practice, there are many causes and triggers of acute exacerbation, with over 80% of AECOPD cases related to bacterial or viral infections (Ko et al., 2016). It has a significant impact on patients’ quality of life, disease progression, mortality rate, and medical costs. Early intervention treatment can help reduce mortality rate, readmission rate, and socio-economic burden (Ismail, 2009; Suissa et al., 2012; Zhou et al., 2015; Bai et al., 2022).

In recent years, with the continuous deepening of research on microbial communities, the relationship between respiratory and intestinal microbiota in COPD and AECOPD patients has also become a research hotspot (Lai et al., 2022; Qu et al., 2022; Russo et al., 2022). After using classical cultivation techniques, it was found that the lower respiratory tract and lungs of healthy individuals are sterile, while the respiratory tract of COPD patients is colonized by opportunistic pathogens (Kahn and Jones, 1987). However, 16S rRNA gene sequencing indicates the presence of a large microbial community in both healthy and diseased states in the lower respiratory tract. Early genetic sequencing research mainly focused on gut microbiota. Recent studies have shown that the respiratory tract, like the intestine, also contains a large number of colonized bacteria. The sputum microbiome of COPD and AECOPD patients is increasingly recognized for its clinical relevance (Yuksel et al., 2023). The use of sputum specimens for respiratory microbiome research has gradually become a clinical research hotspot (Hilty et al., 2010; Erb-Downward et al., 2011). Early biomarkers are a class of biological molecules or cellular features that can assist in diagnosis in the pre- or early stages of disease occurrence. Metabolomics can detect early metabolic changes in the disease by measuring differential metabolites between the disease group and the control group, making it useful for identifying early biomarkers of COPD and AECOPD (Yu et al., 2019).

Despite increasing evidence suggesting the role of microorganisms and related metabolites in COPD and AECOPD, there are still some unresolved issues regarding their clinical relevance (Chen and Qiu, 2019). So far, most studies have focused on the gut microbiome. This study analyzed the respiratory microbiota and serum metabolites using 16S rRNA sequencing and metabolomics techniques, with a focus on the similarities and differences in respiratory microbiota of COPD and AECOPD patients, changes in microbiota and serum metabolites related to disease progression. The purpose is to improve the overall understanding of AECOPD, prevent its acute attack early and alleviate the pain of patients, which is also of great significance to reduce its incidence rate and mortality.

## 2 Materials and methods

### 2.1 Ethical approval and informed consent

This study was approved by the Ethics Committee of the First Affiliated Hospital of Henan University of Traditional Chinese Medicine in accordance with the Helsinki Declaration (2019HL-016-02). Notify patients participating in this study of relevant information and obtain their consent, and sign an informed consent form.

### 2.2 Research subjects and enrollment process

#### 2.2.1 Research subjects

18 COPD patients (COPD group), 36 AECOPD patients (AECOPD group), and 10 healthy volunteers (HC group) admitted to the First Affiliated Hospital of Henan University of Traditional Chinese Medicine from June 2022 to June 2023 were selected as the study subjects. Collect basic and main clinical information of three groups of research subjects, including basic information (age, gender, height, weight, smoking history) and clinical data (previous number and degree of acute exacerbations, comorbidities, blood routine, liver and kidney function, inflammatory indicators, lung function, Modified Medical Research Council respiratory distress scale (mMRC) and GOLD grading, etc.). The basic information and main clinical information of all research subjects are shown in Table 1. The severity of AECOPD, mMRC, and GOLD grading were shown in Supplementary Tables S1–S3.

**Table 1:** Clinical data of healthy volunteers, COPD and AECOPD patients.

	HC *n* = 10	COPD *n* = 18	AECOPD *n* = 36	*p*- value
Age (year)	65.07 ± 6.67	63.56 ± 8.80	66.94 ± 6.44	0.253
Gender (Male %)	80.0	61.1	83.3	0.179
BMI (kg/m^2^)	23.95 ± 3.47	24.97 ± 4.15	23.16 ± 3.54	0.243
Current smoking (%)	46.7	44.4	30.6	0.439
Smoking volume (per year)	110.00 (0, 365.00)	0 (0, 465.00)	328.50 (0, 527.25)	0.323
mMRC scoring	NA	2.00 ± 0.34	2.42 ± 0.87	0.016
CAT scoring	NA	12.11 ± 6.43	15.67 ± 5.25	0.034
FEV1/FVC	67.93 ± 14.60	58.13 ± 11.44	47.57 ± 12.46	<0.01
FEV1% predicted values	78.38 ± 21.91	61.79 ± 26.30	49.43 ± 19.98	<0.01
6MWD	496.60 ± 78.44	371.83 ± 94.75	337.22 ± 112.10	<0.01
Sputum neutrophil proportion (%)	12.45(10.00, 69.33)	43.92 (19.20, 94.91)	87.27 (63.54, 97.31)	<0.01
Allergy history				0.343
Yes	NA	1	7	
No	NA	17	29	
Past history of aggravation				0.336
0–1	NA	7	19	
≥2	NA	11	17	
GLOD grading				<0.01
1	NA	6	1	
2	NA	3	15	
3	NA	8	15	
4	NA	1	5	

#### 2.2.2 Enrollment process

Inclusion criteria: (1) Diagnosis of COPD: According to the Global Initiative for Chronic Obstructive Lung Disease (GOLD) guidelines, after using bronchodilators, all COPD patients have a forced expiratory volume in 1 s (FEv1)/forced vital capacity (FVC) of less than 0.7. (2) AECOPD is defined as acute exacerbation of respiratory symptoms that have not been treated with antibiotics in the previous month prior to enrollment. (3) All patients have no consciousness disorders or hemodynamic disorders.

Exclusion criteria: (1) Exclude individuals with a history of bronchial asthma, allergic rhinitis, or hereditary allergies. (2) Excluding conditions such as pulmonary fibrosis, atelectasis, and pulmonary embolism. (3) Exclude individuals with severe cardiovascular and cerebrovascular diseases or renal failure. (4) Long term oral administration of glucocorticoids or immunosuppressive drugs. (5) Individuals aged over 90 or unable to cooperate with the examination.

### 2.3 Peripheral blood and sputum samples collection

Take 5 mL of fasting venous blood from 7:00 to 9:00 in the morning, centrifuge at 3000 rpm for 10 min, take the upper serum, place it in an EP tube, and store it at −80°C for subsequent metabolite identification. By guiding the research subjects to cough correctly and effectively, collect deep airway sputum spontaneously coughed up as sputum specimens. Collecting the phlegm in a disposable sterile sputum cup. Immediately after packaging, freeze the sample at −80°C for subsequent DNA extraction.

### 2.4 Serum metabolome

Thermo Scientific Hypersil ACE3C18 chromatographic column (150 mm × 3.0 mm × 3 μm) was used to chromatographic analysis. TOF/MS was performed on both positive and negative ion modes. The conditions of chromatography and mass spectrometry, as well as the metabolomics data analysis, referred to Zhang et al. (2023).

### 2.5 Sputum microbiome

#### 2.5.1 DNA extraction and quality inspection

Tiangen (Beijing, China) efficient oral swab genomic DNA extraction kit (DP362) was used for DNA extraction, and follow the instructions for all operations. DNA purity and quality were detected by Nano Drop2000 and 1% agarose gel electrophoresis, respectively.

#### 2.5.2 Construction and quality inspection of DNA library

Illumina’s TruSeq Nano DNA LT Library Prep Kit was used for library construction. After the library construction is completed, take 1 μL of the library and perform a quality check on the library using the Agilent High Sensitivity DNA Kit on an Agilent Bioanalyzer 2,100. Quantify the library using Quant iT PicoGreen dsDNA Assay Kit on Promega QuantiFluor, and the qualified library should have a calculated concentration of 2 nM or higher.

#### 2.5.3 High throughput sequencing

The V3-V4 variable region of 16S rDNA gene was selected as the target for PCR amplification. The universal primers used are as follows: F341 (5’-ACTCC-TACGGGRSGCAGCAG-3′), 806R (5’-GACTACHV-GGGTWTCTAAT-3′). Illumina Novaseq6000 SP PE250 was used to detect the microbial community diversity.

#### 2.5.4 Bioinformatics analysis

Alpha and beta diversity: By randomly selecting a certain number of sequences from each sample, it is possible to predict the total number of species that the sample may contain and the relative abundance of each species at a given sequencing depth, that is, to perform species accumulation curve analysis. Using the flattened ASV/OTU table, called the “QIIME diversity core metrics phylogenetic” or “QIIME diversity core metrics” command based on the presence or absence of the tree file, calculate the Bray Curtis distance matrix, and perform PCoA analysis on these distance matrices. Species composition: By statistically analyzing the feature table after removing singletons, visualize the composition distribution of each sample at the phylum and genus classification levels, and present the analysis results in a bar chart. Species differences and marker species analysis: Venn plots were created using ASV/OTU abundance tables. According to the presence or absence of ASV/OTU in each sample (group), the number of members in each set is counted separately, that is, the number of ASV/OTU unique to each group and shared between groups. Draw a heatmap using the abundance data of the top 20 genera with average abundance. LEfSe analysis: 1. For species that show significant differences through Kruskal-Wallis test, if the abundance of all groups is different, they become the test differential species. 2. Wilcoxon test: After identifying the differentially expressed species, Wilcoxon test can be used to determine the significance of inter group differences. The testing strategy should be consistent with the comparison strategy, and the significantly expressed species are the differentially expressed species. 3. LDA threshold: Perform LDA analysis on the identified differential species to estimate the magnitude of the effect of each differential component abundance on inter group differences. Set the threshold to 3.5, and only differential species that pass this threshold are considered as marker species. Functional potential prediction: Annotate the 16S rRNA gene sequence in the Kyoto Encyclopedia of Genes and Genomes (KEGG) database to predict the function of microbial communities.

### 2.6 Correlation analysis between microbial community and metabolites

The correlation analysis between differential respiratory microbiota and serum metabolites was performed using the spearman algorithm through the genescloud tools.^1^

### 2.7 Statistical methods

All data were processed using SPSS 24.0 statistical software. One way analysis of variance (ANOVA) was used for data that followed a normal distribution, while non parametric tests (Kruskal Wallis) were used for data that did not follow a normal distribution. Measurement data is expressed as mean ± standard deviation (± s). *p* < 0.05 is considered statistically significant.

## 3 Results

### 3.1 Clinical data

The basic information and clinical data of the subjects was shown in Table 1. There were no statistically significant differences among the three groups in terms of age, gender, BMI, current smoking, smoking volume, allergy history and past history of aggravation (*p*-value >0.05). However, the mMRC scoring, CAT scoring and Sputum neutrophil proportion in the AECOPD group were higher than those in the COPD group, and the difference was statistically significant (*p*-value <0.01 or *p*-value <0.05). The common indicators of lung function in COPD group, such as FEV1/FVC, FEV1% predicted values and 6MWD were higher than those in AECOPD group, and the difference was statistically significant (*p*-value <0.01).

### 3.2 Diversity of sputum microbiota

**Figure 1:**
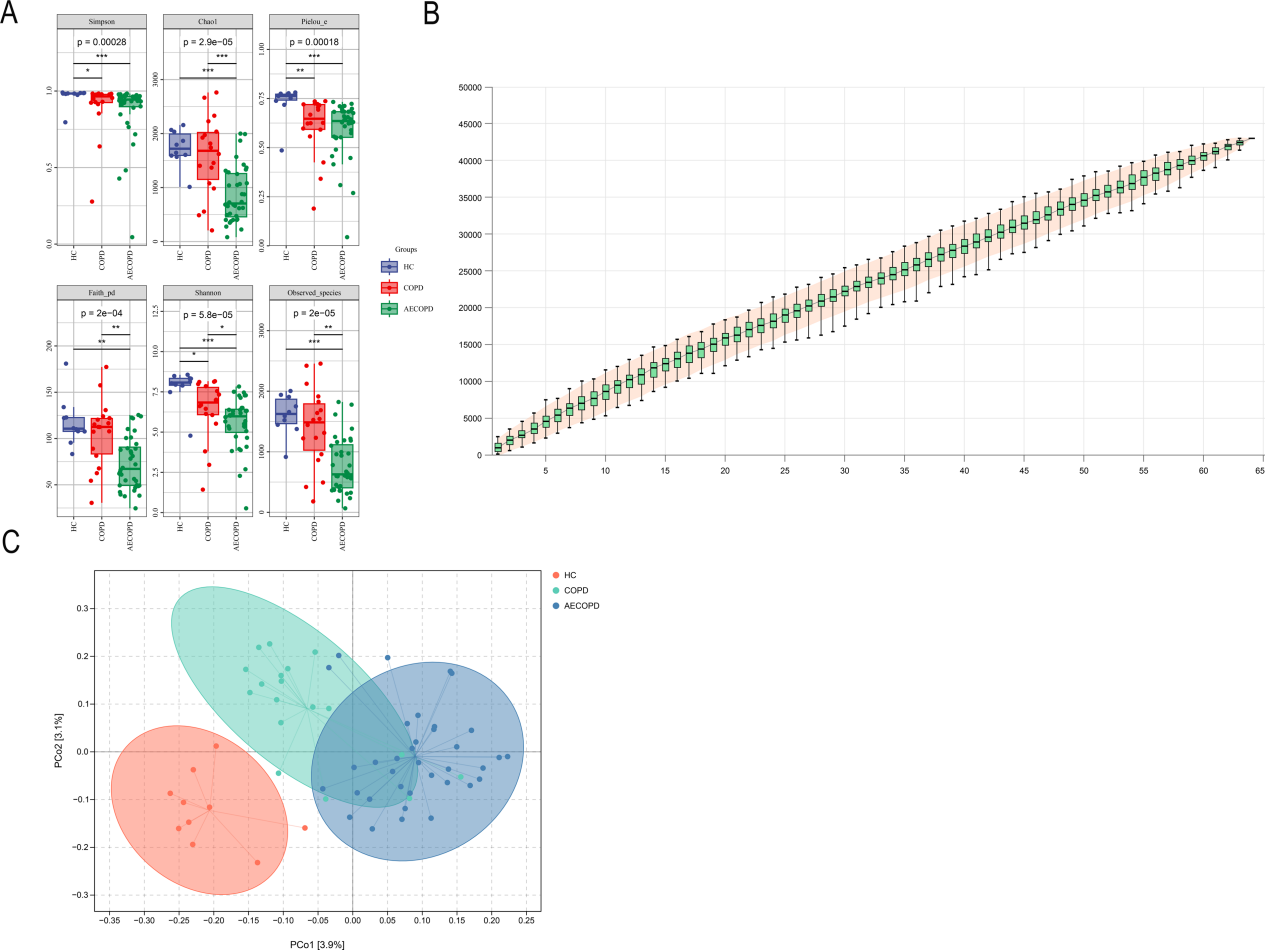
Alpha and beta diversity results. **(A)** Alpha diversity indexes (Simpson, Chao1, Pielou-e, Faith_pd, Shannon and Observed_specific indexes). **(B)** Species cumulative curve. **(C)** Principal co-ordinates analysis.

The results of the alpha diversity of sputum microbiota showed that the Chao1, Observed species, Shannon, Simpson, Faith’s PD and Pielou’s e indexes in HC group were significantly higher than those in AECOPD group. The Shannon, Simpson and Pielou’s e indices in the alpha diversity indexes of HC group are significantly higher than those of COPD group. The Chao1, Faith’s PD, Shannon and Observed species indices in the alpha diversity index of COPD group were significantly higher than those of AECOPD group (Figure 1A). As the sample size increases, the curve will show a sharp upward trend. When the sample size reaches 60, the total number of ASV/OTUs in the community will no longer significantly increase with the addition of new samples, and the curve will also tend to flatten, indicating that 66 sample sizes are sufficient to reflect the species composition of the community (Figure 1B). The projection distance of each sample among the three groups on the coordinate axis is relatively far, indicating that the community composition of each sample among the groups is not similar in the corresponding dimension, while the projection distance of each sample within the three groups on the coordinate axis is relatively close, indicating that the community composition of each sample within the groups is similar in the corresponding dimension (Figure 1C).

### 3.3 Composition and distribution of sputum microbiota

**Figure 2:**
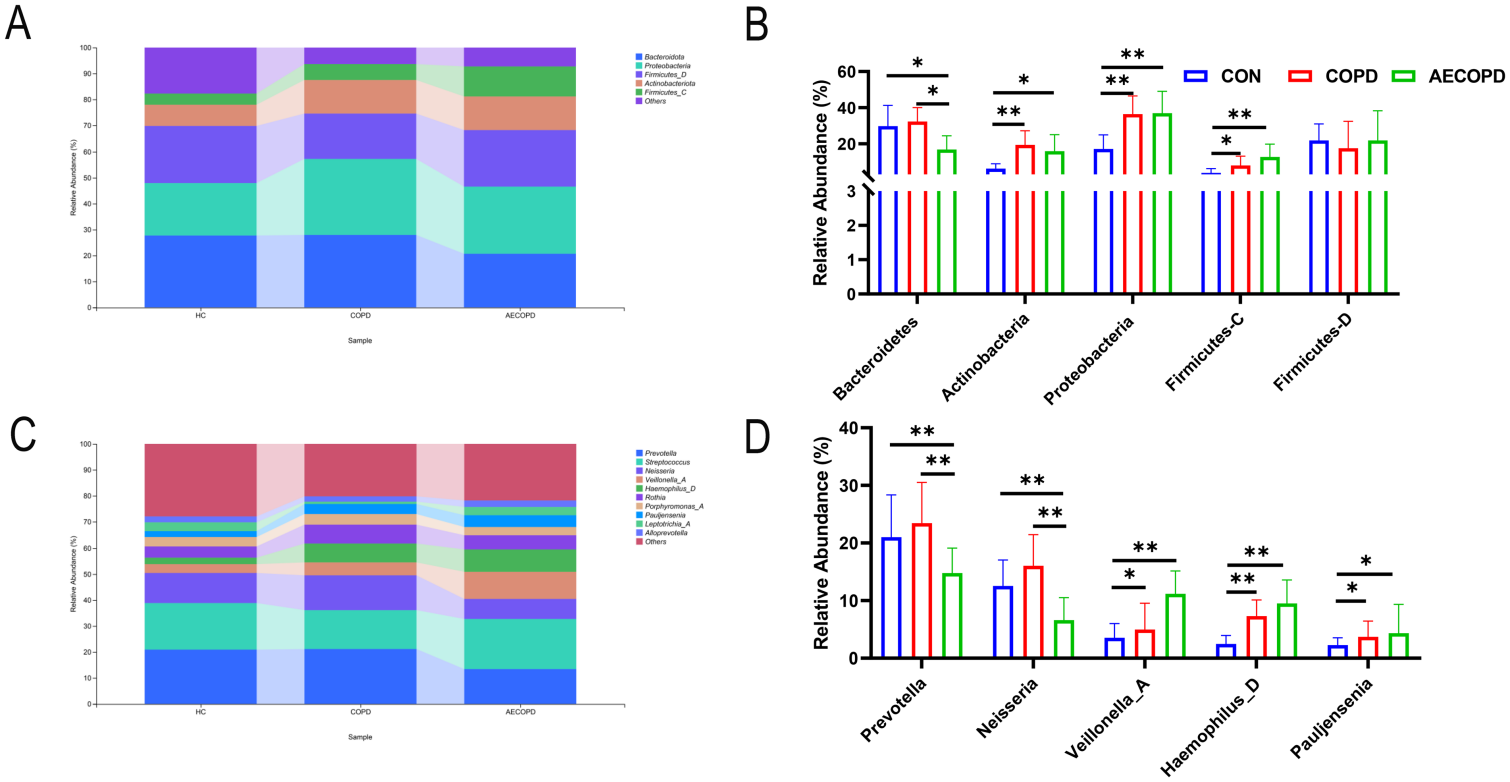
Composition and distribution of microbial communities at the phylum and genus levels. **(A,B)** Composition and distribution of microbial communities at the phylum level. **(C,D)** Composition and distribution of microbial communities at the genus level.

At the phylum level, there was no significant difference in the relative abundance of Bacteroidetes in sputum of healthy individuals and COPD patients, while the relative abundance of Bacteroidetes in sputum of healthy individuals and COPD patients was significantly higher than that in AECOPD patients. In addition, the relative abundance of Actinobacteria, Proteobacteria and Firmicutes-C in sputum of healthy individuals was significantly lower than that of COPD patients and AECOPD patients, while the relative abundance of Proteobacteria in sputum of COPD patients was not significantly different from that of AECOPD patients. There was no significant difference in the relative abundance of Firmicutes-D among the three groups (Figures 2A,B). At the genus level, there was no significant difference in the relative abundance of *Prevotella* and *Neisseria* in sputum of healthy individuals and COPD patients, while the relative abundance of *Prevotella* and *Neisseria* in sputum of healthy individuals and COPD patients was significantly higher than that in AECOPD patients. In addition, the relative abundance of *Veillonella_A*, *Haemophilus_D*, and *Pauljensonia* in sputum of healthy individuals was significantly lower than that of COPD and AECOPD patients, while the relative abundance of *Veillonella_A*, *Haemophilus_D* and *Pauljensonia* in sputum of COPD patients was not significantly different from that of AECOPD patients. There was no significant difference in the relative abundance of *Streptococcus* among the three groups (Figures 2C,C).

### 3.4 Analysis of species differences and marker species

**Figure 3:**
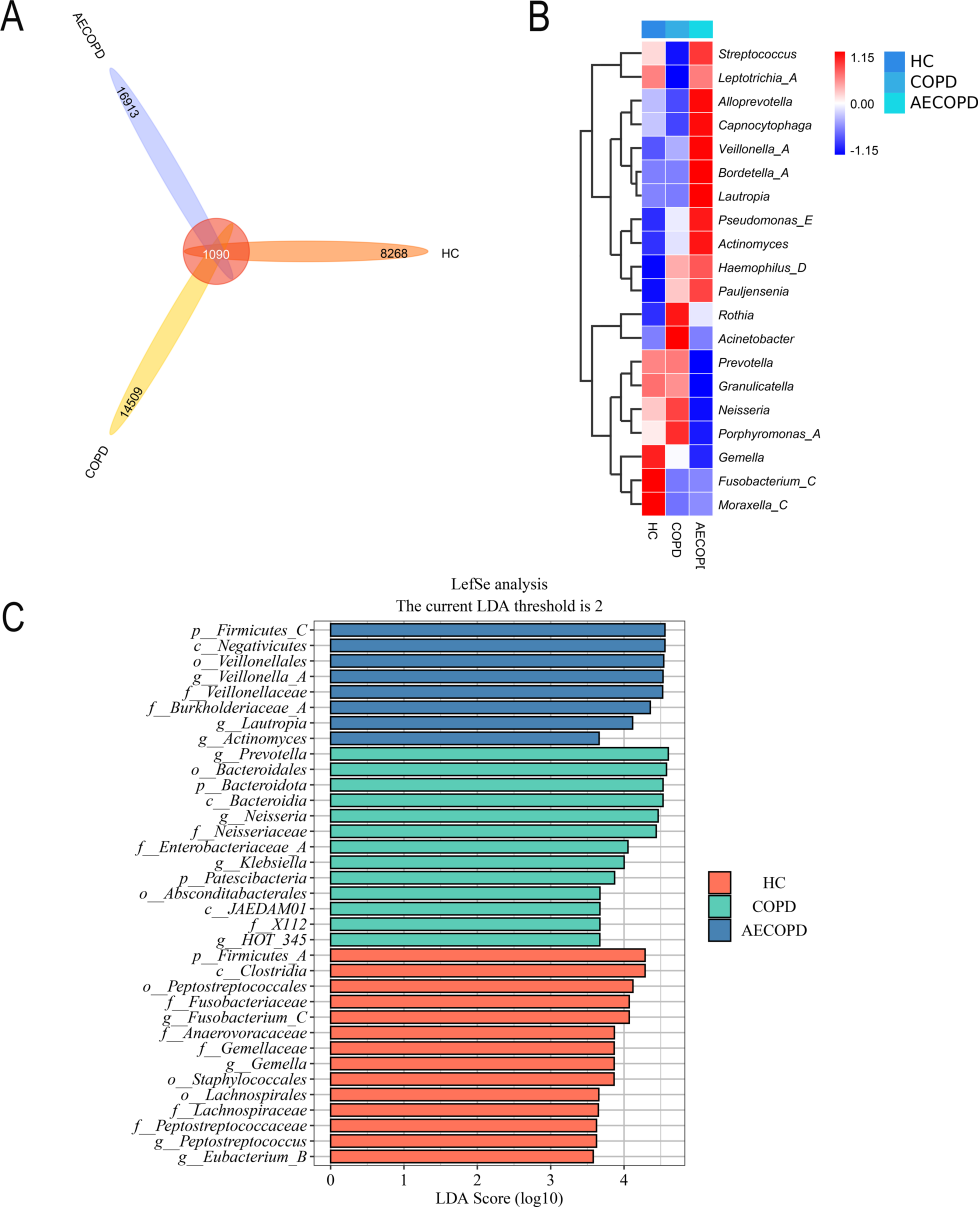
Species differences and marker species. **(A)** Venn diagram based on ASV level. **(B)** Species composition heatmap (Genus level). **(C)** LefSe analysis.

The Venn diagram results showed that the HC group, COPD group and AECOPD group had 8,268, 14,509, and 16,913 ASVs, respectively, and shared 1,090 ASVs (Figure 3A). The species composition heatmap results showed that both the COPD group and the AECOPD group significantly increased the relative abundance of *Rothia*, *Haemophilus_D* and *Pauljensonia*, while significantly decreased the relative abundance of *Fusobacterium* and *Moraxella* compared with the HC group. The AECOPD group significantly increased decreased the relative abundance of *Streptococcus*, *AlloPrevotella*, *Capnocytophaga*, *Veillonella*, *Bordetella*, *Lautropia*, *Pseudomonas_E*, *Actinobacteria, Haemophilus_D* and *Pauljensenia*, while significantly decreased the relative abundance of *Prevotella*, *Granulicatella*, *Neisseria*, *Porphyromonas_A* and *Gemella* compared with the COPD group (Figure 3B). The LEfSe analysis results showed that compared with the COPD group and AECOPD group, p_Firmicutes, c_Clostridia and o_Peptostatocccales were significantly different microbial communities in the HC group. Compared with the HC group and AECOPD group, *g_Prevotella*, o_Bacteroidales and p_Bacteroidota were significantly different microbial communities in the COPD group; Compared with the HC group and COPD group, p_Firmicutes, c_Negativicutes and o_Veillonellales were significantly different microbial communities in the AECOPD group (Figure 3C).

### 3.5 Prediction of microbial community functional potential

**Figure 4:**
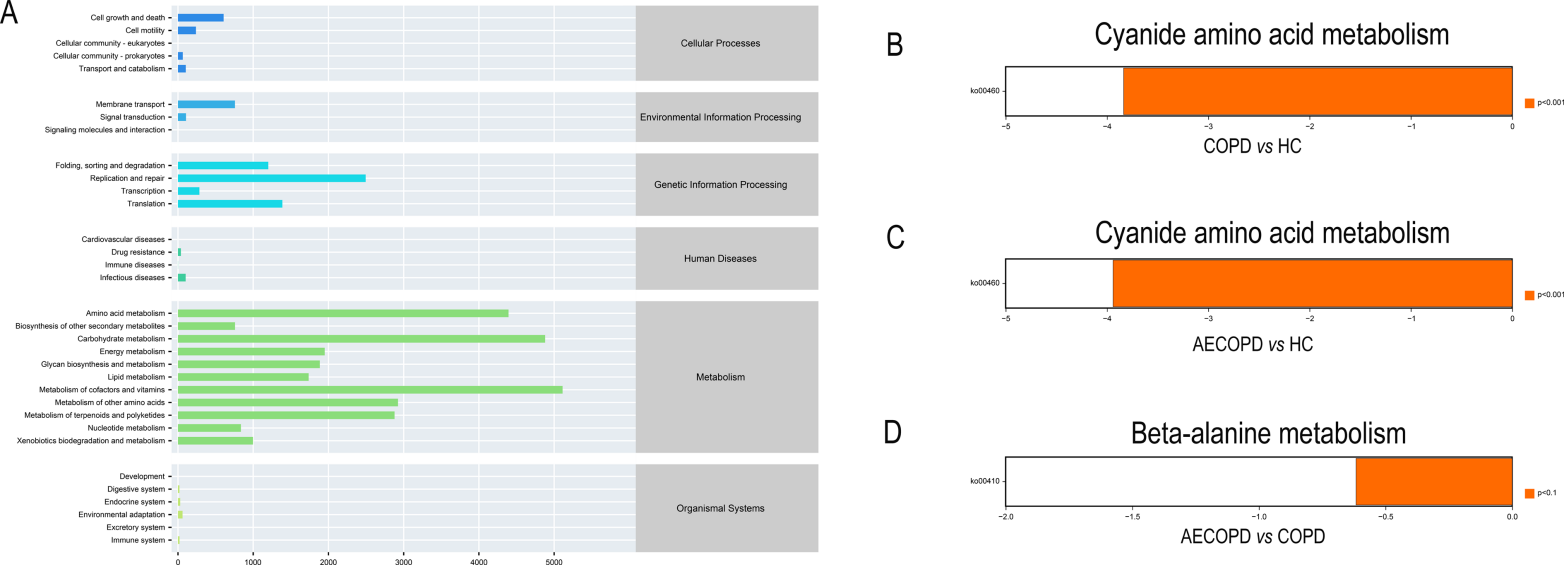
PICRUSt2 Functional potential prediction. **(A)** Microbial communities participate in the metabolic pathways of KEGG. **(B–D)** Significant differences in metabolic pathways among the three groups.

The KEGG metabolic pathway results showed that the microbiota is mainly involved in metabolism (metabolism of cofactors and vitamins, carbohydrate metabolism and amino acid metabolism), genetic information processing (replication and repair, translation and folding, sorting and degradation) and environmental information processing (membrane transport and signal transmission) (Figure 4A). The analysis of metabolic pathway differences showed that both the COPD group and the AECOPD group significantly downregulated the KO00460 pathway (cyanide amino acid metabolism) compared with the HC group (Figures 4B,C). The AECOPD group significantly downregulated the KO00410 pathway (*β*-alanine metabolism) compared with the COPD group (Figure 4D).

### 3.6 Quality control, identification, and analysis of metabolites

**Figure 5:**
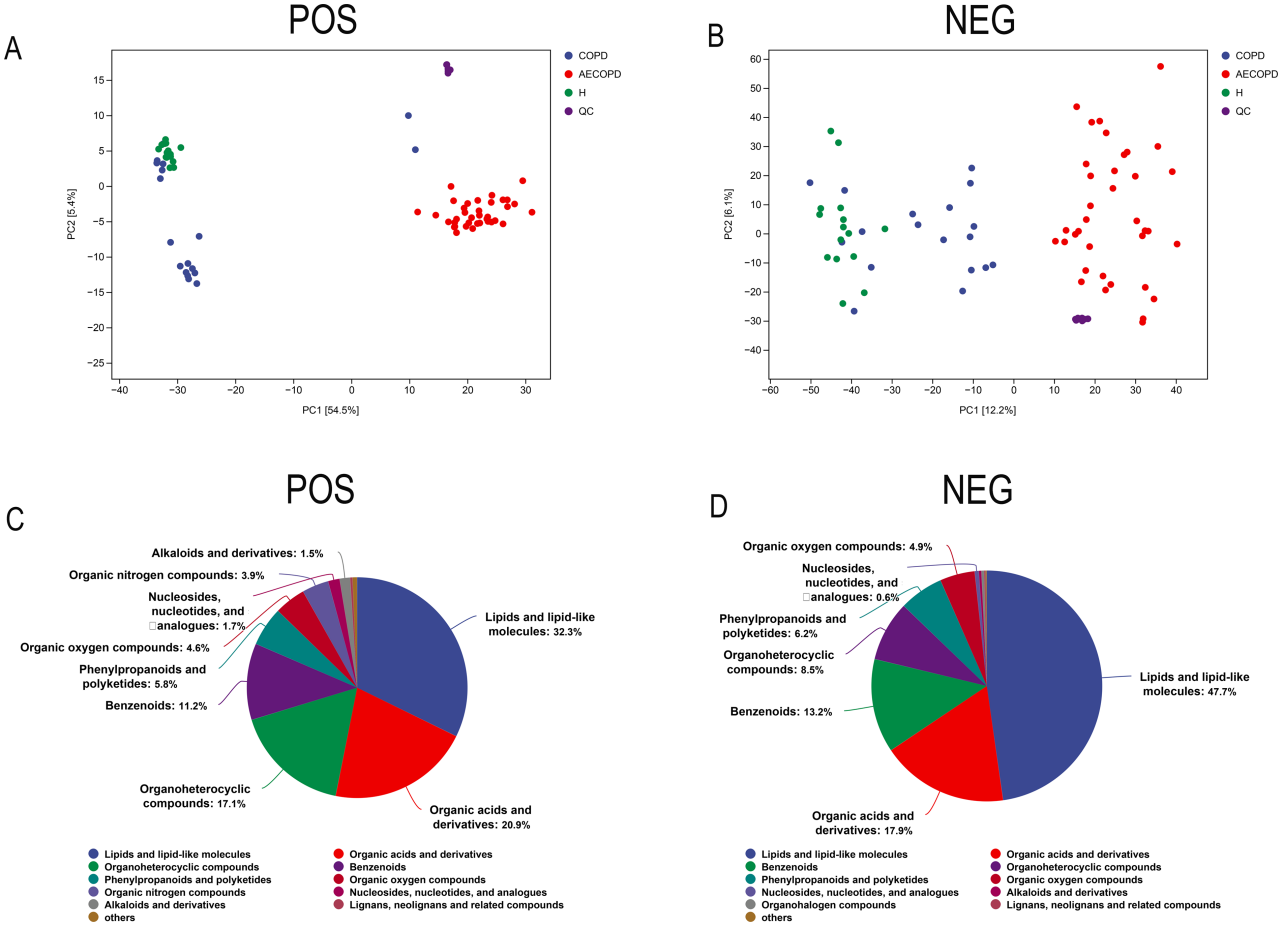
Metabolome data quality control and overall metabolite identification. **(A)** PCA analysis of the overall samples in positive ion mode. **(B)** PCA analysis of the overall samples in negative ion mode. **(C)** Identification results of metabolites in positive ion mode. **(D)** Identification results of metabolites in negative ion mode.

**Figure 6:**
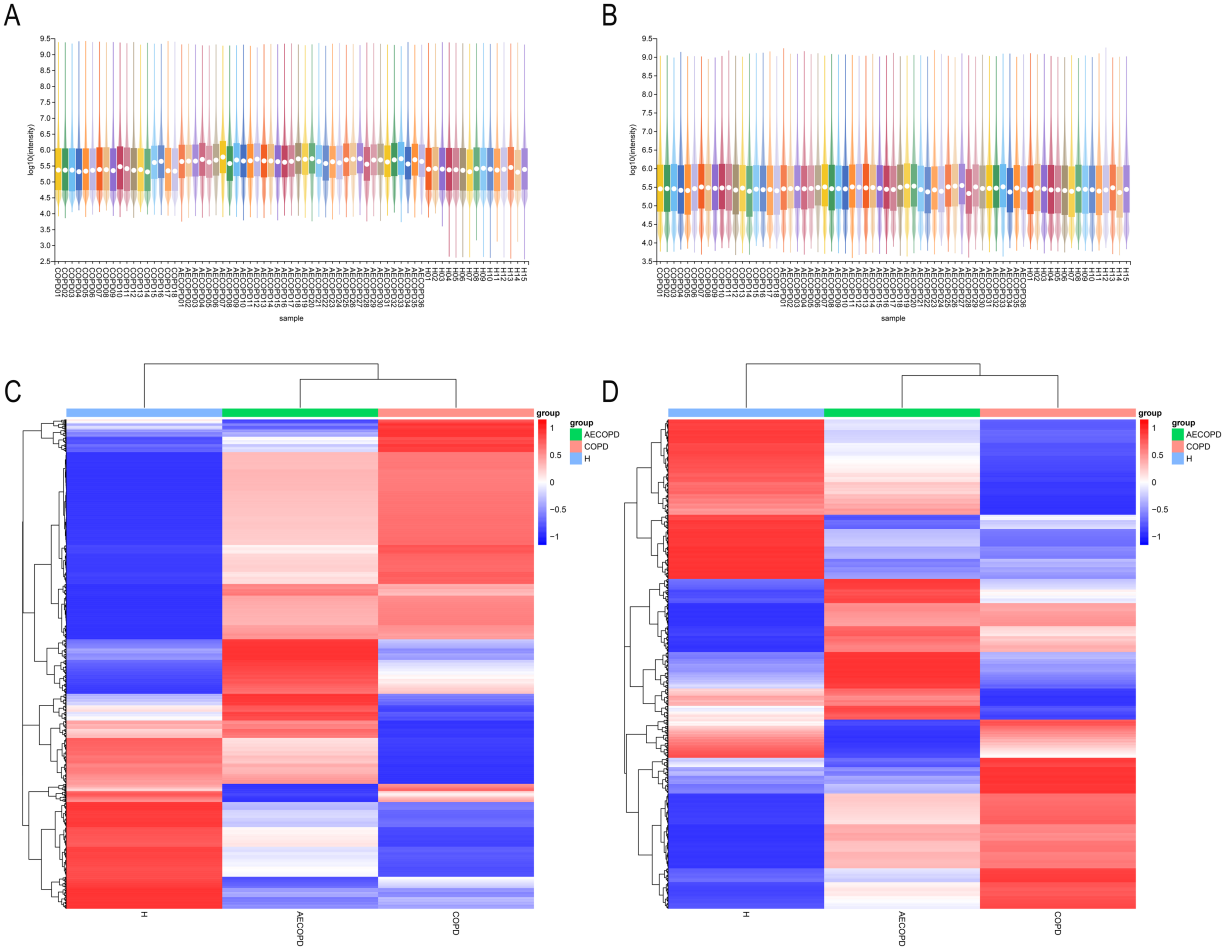
Expression abundance analysis. **(A)** Statistical results of expression abundance of metabolites in each sample under positive ion mode. **(B)** Statistical results of expression abundance of metabolites in each sample under negative ion mode. **(C)** Overall material clustering heatmap in positive ion mode. **(D)** Overall material clustering heatmap in negative ion mode.

The PCA results of quality control show that the distance between samples in the QC group is close, indicating that the higher the stability of the method, the better the data quality, and the data is reliable. In addition, the sample dispersion among the HC group, COPD group and AECOPD group was relatively large, indicating significant differences among the groups. The clustering of samples within the group, with close proximity indicating high similarity among samples (Figures 5A,B). In the positive ion mode, lipids and lipid like molecules (32.3%), organic acids and derivatives (20.9%), organoheterocyclic compounds (17.1%), benzoids (11.2%), phenylpropanoids and polyketides (5.8%), organic oxygen compounds (4.6%), organic nitrogen compounds (3.9%), nucleosides, nucleotides, and analogs (1.7%), alkaloids and derivatives (1.5%), lignans, neolignans, and related compounds (0.3%) were detected (Figure 5C). In negative ion mode, lipids and lipid like molecules (47.7%), organic acids and derivatives (17.9%), benzoids (13.2%), organoheterocyclic compounds (8.5%), phenylpropanoids and polyketides (6.2%), organic oxygen compounds (4.9%), nucleosides, nucleotides, and analogs (0.6%), alkaloids and derivatives (0.3%), organohalogen compounds (0.3%), lignans, neolignans and related compounds (0.1%) were detected (Figure 5D). In positive and negative ion modes, the expression abundance of metabolites was statistically analyzed, and the results showed that the expression abundance patterns of all substances in each sample were relatively close, indicating that the overall substance spectra of the samples were closer, that is, more similar (Figures 6A,B). Display the abundance changes of the same substance in different samples through a heatmap, with each column representing a group and each row representing a substance. In both positive and negative ion modes, the same metabolite has a high metabolic level in the HC group, a lower metabolic level in the COPD and AECOPD groups, a low expression metabolite in the HC group, and a high expression metabolite in the COPD and AECOPD groups (Figures 6C,D).

### 3.7 Differential metabolites analysis

**Figure 7:**
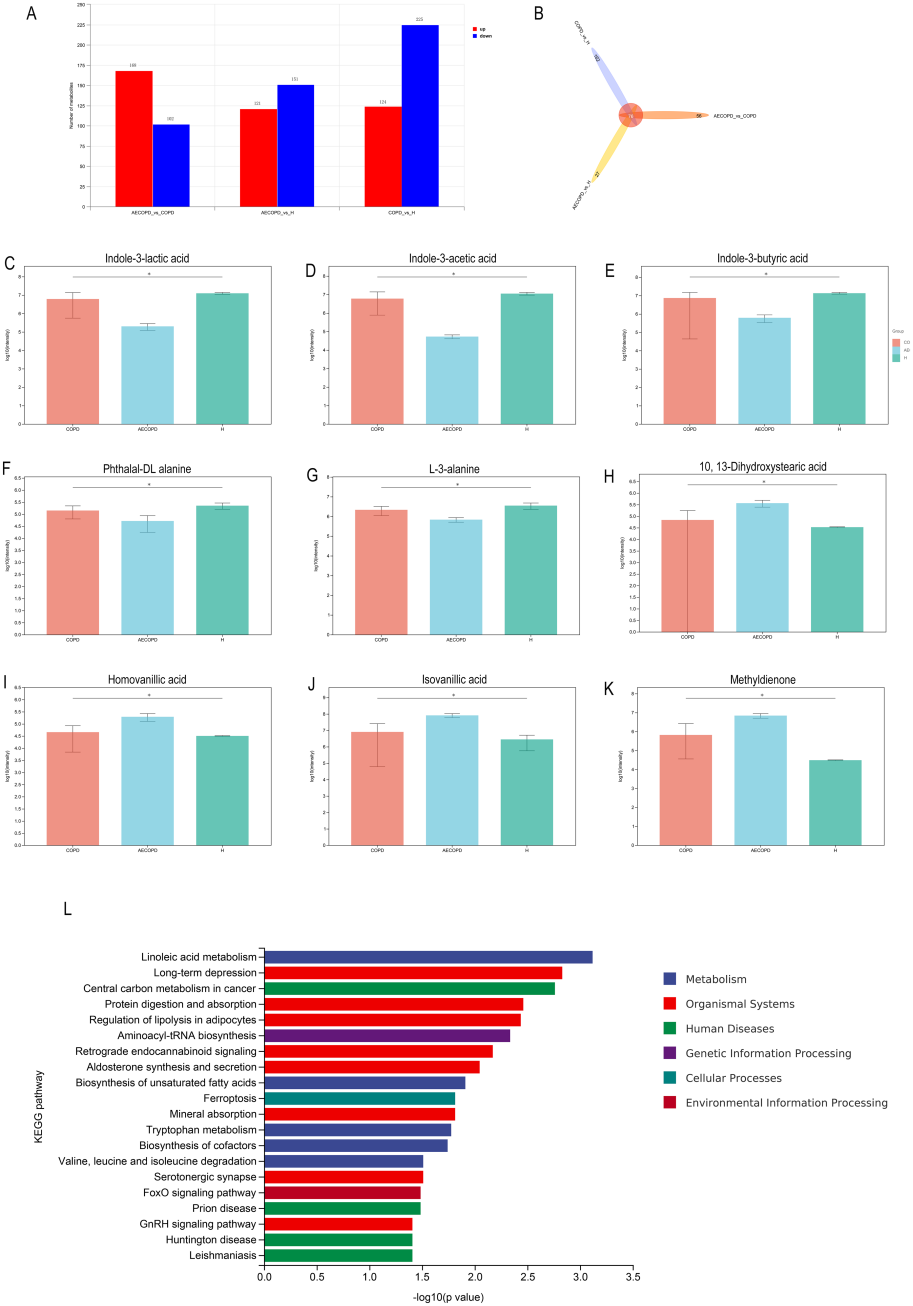
Differential metabolite analysis. **(A)** Differential substance screening in positive and negative ion merging mode. **(B)** Differential substance Venn analysis in positive and negative ion merging mode. **(C–K)** Metabolites with significant differences in AECOPD compared to HC and COPD in positive and negative ion combination mode. **(L)** KEGG enrichment analysis of differential substances in positive and negative ion merging mode.

In Merge mode (positive and negative ion merging mode), there are a total of 270 differential metabolites between AECOPD and COPD, including 168 upregulated metabolites and 102 downregulated metabolites; There are a total of 272 differential metabolites between AECOPD and HC, including 121 upregulated metabolites and 151 downregulated metabolites; There are a total of 349 differential metabolites between COPD and HC, including 124 upregulated metabolites and 225 downregulated metabolites (Figure 7A). In Merge mode, the Venn plot results showed that AECOPD vs. COPD, AECOPD vs. HC, and COPD vs. HC independently shared 56, 27, and 162 metabolites, respectively, and collectively shared 76 metabolites (Figure 7B). Further analysis of differential metabolites revealed that AECOPD significantly downregulated the relative abundance of metabolites such as indole-3-lactic acid, indole-3-acetic acid, indole-3-butyric acid, and L-3-alanine and phthalal-DL alanine compared to the COPD and HC groups, and significantly upregulated the relative abundance of metabolites such as isovanillic acid, 10,13-dihydroxystearic acid, homovanillic acid and methyldienone (Figures 7C–K). The KEGG metabolic pathway results showed that differential metabolites were mainly annotated to metabolic pathways such as linoleic acid metabolism, long-term depression, central carbon metabolism in cancer, protein digestion and absorption, regulation of lipolysis in adipocytes and aminoacyl-tRNA biosynthesis (Figure 7L).

### 3.8 Correlation analysis between microbial community and metabolites

**Figure 8:**
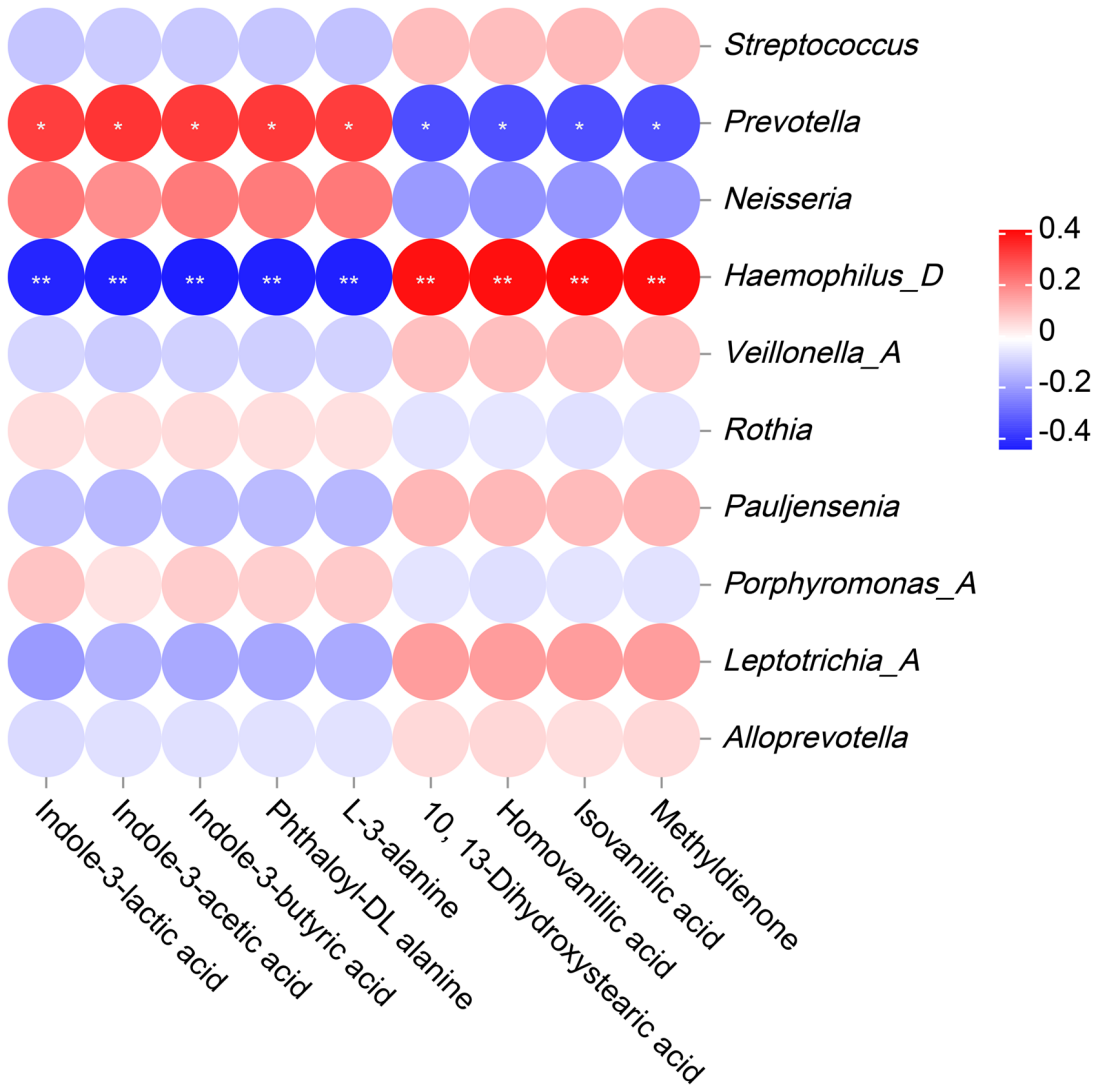
The Correlation analysis between microbiota and metabolites. The legend displays the correlation coefficient values, where red represents positive correlation, blue represents negative correlation, and the depth of color indicates the strength of correlation. * or ** indicates significant correlation between microorganism and metabolite (*p* < 0.05 or *p* < 0.01).

The correlation analysis results revealed that *Prevotella* was significantly positively correlated with indole-3-lactic acid, indole-3-acetic acid, indole-3-butyric acid, phthaloyl-DL alanine and L-3-alanine, and significantly negatively correlated with 10, 13-Dihydroxystearic acid, homovanillic acid, isovanillic acid and methyldienone. However, *Haemophilus_D* showed completely opposite results from *Prevotella*, with significant negative correlations with indole-3-lactic acid, indole-3-acetic acid, indole-3-butyric acid, phthaloyl-DL alanine, and L-3-alanine, and significant positive correlations with 10, 13-Dihydroxystearic acid, homovanillic acid, isovanillic acid, and methyldienone. (Figure 8).

## 4 Discussion

The respiratory microbiota plays an important role in maintaining normal airway immune responses and balancing the relationship among the host, microbiota, and environment. It not only protects the host from pathogen invasion (Budden et al., 2019), but also regulates the immune system and nutrient absorption (Shah et al., 2021). The role of microbial infections in the occurrence and development of COPD is a hot topic in respiratory microbiome research (Sethi, 2014). According to reports, respiratory microbiota colonization increases the risk of chronic obstructive pulmonary disease (COPD) lung infection and poor prognosis (O'Shaughnessy et al., 2023; Li et al., 2024). As microbiota colonization induces the expression of inflammation in the body, it triggers immune inflammatory responses, induces the occurrence of AECOPD, promotes disease progression, and damages lung function (Leung et al., 2017; Liu et al., 2021). In recent years, research on respiratory microbiota has focused on postoperative and critical care areas (Wang et al., 2021; Zheng et al., 2023). The role of respiratory microbiota in the occurrence and development of AECOPD needs further clarification. Therefore, this study aims to provide references for clinical guidance of treatment strategies, prediction of acute exacerbation risk, evaluation of disease prognosis, and guidance of microbial therapy by assessing the diversity of respiratory microbiota and serum metabolites in healthy individuals, COPD patients, and AECOPD patients.

The difference in alpha diversity of respiratory microbiota among the three research groups is mainly characterized by the lowest in the AECOPD group and the highest in the healthy population, other studies have also found that the diversity of respiratory microbiota in patients with acute exacerbation of chronic obstructive pulmonary disease (COPD) is significantly reduced compared to stable patients (Huang et al., 2014; Sun et al., 2020; Su et al., 2022). These results suggest that the occurrence and development of AECOPD seem to be related to the reduction of microbial diversity. The study by Wang et al. is similar to the results of this experiment, which also found that compared to the stable phase, the microbiota of AECOPD patients showed a decrease in microbial composition toward the phylum Bacteroidetes and an increase toward the Proteobacteria (Wang et al., 2016). Pragman et al. by sequencing samples from bronchoalveolar lavage fluid of moderate and severe COPD patients, found that the microbial community structure in the lungs of these two patient groups had changed, and Firmicutes, Proteobacteria and Actinobacteria were abundant in the lower respiratory tract of these patients (Pragman et al., 2012). PCoA analysis shows significant differences between healthy individuals and COPD patients. The composition of respiratory microbiota in stable COPD and AECOPD patients is more similar and there is significant overlap. Considering that the respiratory microbiota is susceptible to interference from multiple factors, this result may be due to the small sample size in stable COPD and cross-sectional studies. Subsequent research needs to consider adding samples at different time points and expanding the sample size.

The above research indicates that the composition and diversity of respiratory microbiota community structure show certain differences at different stages of the disease. With the occurrence of acute attacks, the alpha diversity of respiratory microbiota decreases, specifically manifested as changes in the abundance of existing species in the respiratory tract, with few new species being discovered. Studies have shown that COPD, AECOPD, and healthy populations have similar species composition in their communities, but the difference lies in the relative abundance of microorganisms between the two groups (Diao et al., 2018). Therefore, the reason for this result may be the proliferation of pathogenic microorganisms in the respiratory tract of patients with acute exacerbation, especially the increase in the number of pathogenic Proteobacteria. At the genus level, this is manifested as a decrease in the relative abundance of *Prevotella* and *Neisseria*, accompanied by an increase in the relative abundance of *Haemophilus_D*, but rarely the emergence of new species, resulting in a decrease in microbial diversity and evenness. The relative abundance of *Prevotella* and *Neisseria* in the respiratory tract of COPD patients is significantly higher than that of AECOPD patients, while their relative abundance of and *Haemophilus_D* is significantly lower than that of AECOPD patients. *Neisseria* and *Prevotella* are one of the important members of the respiratory microbiota, widely distributed in the oral cavity, respiratory tract, and other parts, with a relatively large number. As a symbiotic bacterium, *Prevotella* plays an important role in maintaining the balance of respiratory microbiota. Research has shown that the increase in abundance of certain species in *Prevotella*, such as melanogenic *Prevotella*, is associated with a decrease in infection with pathogens such as *Streptococcus pneumoniae*, indicating that *Prevotella* may be an important part of respiratory immune protection. They work together with other microorganisms to resist the invasion of foreign pathogens and protect respiratory health. This study found that the *Prevotella* and *Neisseria* in AECOPD patients were lower than those in COPD patients, suggesting that their reduction is related to the deterioration of severe COPD. Therefore, it is believed that *Prevotella* and *Neisseria* may play a beneficial role in chronic obstructive pulmonary disease.

In addition, compared with the healthy population, the relative abundance of *Pseudomonas* and *Haemophilus_D* in COPD and AECOPD increased significantly, moreover the relative abundance of these microbiota in the AECOPD group was higher than that in the COPD group. Millares et al.’s study also found that in COPD patients, the relative abundance of *Pseudomonas* increased with the degree of airflow restriction (Millares et al., 2019). *Pseudomonas* is a common opportunistic pathogen belonging to the Proteobacteria. Based on the changes in relative abundance of bacteria among the three groups, it can be inferred that the changes in the respiratory microbiome associated with severity are due to a decrease in specific bacterial genera, which may be replaced by *Pseudomonas* (Nakamoto et al., 2019). Sethi et al. reported that inflammatory cells such as neutrophils and lymphocytes in the sputum of COPD patients infected with *Haemophilus parainfluenzae* were significantly higher than those in COPD patients without infection, confirming the association between *Haemophilus* and COPD and its acute exacerbations (Sethi and Murphy, 2001). In patients with AECOPD, the composition of the respiratory tract microbial community changes significantly. The numbers of common pathogenic bacteria such as *Haemophilus influenzae*, *Streptococcus pneumoniae*, and *Pseudomonas aeruginosa* increase (Euba et al., 2017; Leung et al., 2017; Rodrigo-Troyano et al., 2016). This imbalance in the microbial community may lead to a decline in local immune function of the respiratory tract, increase the risk of infection, and thus induce AECOPD. After pathogenic bacteria invade the respiratory tract, they activate the host’s immune system and trigger an inflammatory response. Inflammatory cells such as neutrophils and macrophages release multiple inflammatory mediators, leading to respiratory tract mucosal edema, increased mucus secretion, airway narrowing, and exacerbation of symptoms such as dyspnea (Ko et al., 2016; Hogea et al., 2020; Ritchie and Wedzicha, 2020).

Through serum metabolomics research, it has been found that amino acid metabolism and lipid metabolism are closely related to AECOPD. Research has shown that metabolite scores based on the weighted sum of serum metabolite concentrations of pyruvate, isoleucine, L-methylhistidine, and glutamine are associated with an increased risk of AECOPD (Peng et al., 2023). Labaki et al. also found that low concentrations of tryptophan, isoleucine, valine, and branched chain amino acids were independently associated with the incidence of acute exacerbation of respiratory symptoms within 1 year (Labaki et al., 2019). In addition to amino acid metabolism, lipid metabolism has always been a focus of metabolomics. A recent study further found that the glycerophospholipid metabolic pathway is significantly downregulated in patients with severe COPD, which is associated with the severity of the disease (Zhu et al., 2020). In addition, it was found that the decreased expression of three phospholipids, lysophosphatidylcholine, lysophosphatidylethanolamine, and phosphatidylinositol, may be related to AECOPD (Gai et al., 2021).

The changes in amino acid metabolism and lipid metabolism are closely related to AECOPD, but due to differences in sample size and source among various studies, the above research conclusions still need to be further validated by expanding the sample size or increasing the types of samples. Zhang et al. used LC–MS technology to detect serum samples from 60 AECOPD patients and found significant differences in metabolites such as lactate, succinic acid, and glucose between the two groups compared to healthy individuals. Additionally, AECOPD patients exhibited specific changes in amino acid, energy, and fat metabolism (Zhang et al., 2020). Yang et al. found through research that compared with stable COPD patients, the levels of serum 3-hydroxybutyric acid, lysine, glutamate, glutamine, and pyruvate in AECOPD patients were significantly increased, while the levels of valine, leucine, isoleucine, VLDL, and LDL were significantly decreased. The levels of serum pyruvate, 3-hydroxybutyric acid, lysine, glutamate, and glutamine in AECOPD patients were significantly increased, while the levels of valine, leucine, isoleucine, alanine, choline, 1-methylhistidine, histidine, and others were significantly decreased (Yang et al., 2016). The correlation analysis results showed a significant negative correlation between *Haemophilus* and alanine. *Haemophilus* can express an enzyme UDP-N-acetylmuramylalanine ligase (MurC), which is an enzyme that catalyzes the addition of L-alanine to the UDP-acetylmuramyl nucleotide precursor in *Haemophilus*. This enzyme is a prerequisite for the biosynthesis of peptidoglycan in *Haemophilus* (Mol et al., 2003; Deva et al., 2006; Isa, 2020). That is to say, the overproliferation of *Haemaphilus* requires more alanine for the biosynthesis of peptidoglycan in the bacteria itself, which may be one of the important factors causing amino acid (alanine) metabolism disorders in the body. Therefore, alanine may be used as a biomarker for AECOPD for disease diagnosis, monitoring, and prognosis evaluation. However, the role of abnormal alanine metabolism in the occurrence and development of AECOPD requires further research. Microorganisms and metabolites play important mechanistic and functional roles in the occurrence and development of AECOPD. In-depth study of changes in microbial communities and metabolites will help better understand the pathogenesis of AECOPD and provide new ideas and methods for the diagnosis, treatment, and prevention of the disease.

## 5 Conclusion

In summary, the structural characteristics of respiratory microbiota in COPD and AECOPD patients are different from those in healthy populations, and their microbiota diversity decreases when acute exacerbations occur. Changes in respiratory microbiota are related to pulmonary function, mMRC scoring, CAT scoring and GLOD grading, but not significantly correlated with smoking, gender, age, and some other clinical indicators. In addition, both the predicted microbial community function and metabolomics results indicate that the onset of AECOPD is mainly related to energy and amino acid metabolism disorders, especially alanine metabolism. Subsequent longitudinal studies with multiple centers and large samples are needed, while integrating bacteria, viruses, and fungi into a holistic study to reveal new diagnostic tools and define new diagnosis and treatment models. This will be of great significance for the clinical diagnosis, individualized standardized diagnosis and treatment, and pulmonary function rehabilitation of COPD/AECOPD patients.

## Data Availability

The datasets presented in this study can be found in online repositories. The names of the repository/repositories and accession number(s) can be found at: https://www.ncbi.nlm.nih.gov/, PRJNA1148836.

## Footnotes

^1^https://www.genescloud.cn
