# Supplementary material for: Exploring the potential role of microbiota and metabolites in acute exacerbation of chronic obstructive pulmonary disease
Source: Front Microbiol. 2024 Oct 16;15:1487393. doi: 10.3389/fmicb.2024.1487393 (PMC11526122; doi:10.3389/fmicb.2024.1487393)
Supplement: Supplementary file 1 [file Table_1.pdf]

Table S1 The severity of AECOPD

Classification	Definition
Mild	Treatment with short acting bronchodilators alone
Moderate	Use short acting bronchodilators and antibiotics, with or without oral corticosteroids
Severe	The patient requires hospitalization or emergency or ICU treatment
