# Supplementary material for: Exploring the potential role of microbiota and metabolites in acute exacerbation of chronic obstructive pulmonary disease
Source: Front Microbiol. 2024 Oct 16;15:1487393. doi: 10.3389/fmicb.2024.1487393 (PMC11526122; doi:10.3389/fmicb.2024.1487393)
Supplement: Supplementary file 2 [file Table_2.pdf]

Table S2 Modified Medical Research Council dyspnea scale, mMRC

mMRC evaluation level	Severity
Level 0	Difficulty breathing only occurs during intense physical activity
Level 1	Shortness of breath when walking briskly on flat ground or climbing small slopes on foot
Level 2	Due to shortness of breath, walking on flat ground is slower than peers or requires stopping to rest
Level 3	After walking on flat ground for about 100 m or a few minutes, you need to stop and catch your breath
Level 4	Due to severe breathing difficulties, unable to leave home, or experiencing breathing difficulties while putting on or taking off clothes
