# Supplementary material for: Exploring the potential role of microbiota and metabolites in acute exacerbation of chronic obstructive pulmonary disease
Source: Front Microbiol. 2024 Oct 16;15:1487393. doi: 10.3389/fmicb.2024.1487393 (PMC11526122; doi:10.3389/fmicb.2024.1487393)
Supplement: Supplementary file 3 [file Table_3.pdf]

Table S3 Modified Medical Research Council dyspnea scale, mMRC

GOLD Grading	Severity	Pulmonary function grading (after using bronchodilators)
Level 1	Mild	FEVi accounts for ≥ 80% of the predicted value
Level 2	Moderate	50% ≤ FEVi% < 80% of predicted value
Level 3	Severe	30% ≤ FEVi% < 50% of predicted value
Level 4	Extremely severe	FEVi accounts for < 30% of the predicted value
